# GAMER-MRI in Multiple Sclerosis Identifies the Diffusion-Based Microstructural Measures That Are Most Sensitive to Focal Damage: A Deep-Learning-Based Analysis and Clinico-Biological Validation

**DOI:** 10.3389/fnins.2021.647535

**Published:** 2021-04-06

**Authors:** Po-Jui Lu, Muhamed Barakovic, Matthias Weigel, Reza Rahmanzadeh, Riccardo Galbusera, Simona Schiavi, Alessandro Daducci, Francesco La Rosa, Meritxell Bach Cuadra, Robin Sandkühler, Jens Kuhle, Ludwig Kappos, Philippe Cattin, Cristina Granziera

**Affiliations:** ^1^Translational Imaging in Neurology (ThINk) Basel, Department of Biomedical Engineering, University Hospital Basel and University of Basel, Basel, Switzerland; ^2^Neurologic Clinic and Policlinic, Departments of Medicine, Clinical Research and Biomedical Engineering, University Hospital Basel and University of Basel, Basel, Switzerland; ^3^Research Center for Clinical Neuroimmunology and Neuroscience Basel, University Hospital Basel and University of Basel, Basel, Switzerland; ^4^Division of Radiological Physics, Department of Radiology, University Hospital Basel, Basel, Switzerland; ^5^Department of Computer Science, University of Verona, Verona, Italy; ^6^Signal Processing Laboratory (LTS5), Ecole Polytechnique Fédérale de Lausanne, Lausanne, Switzerland; ^7^CIBM Center for Biomedical Imaging, Lausanne, Switzerland; ^8^Department of Radiology, Lausanne University Hospital and University of Lausanne, Lausanne, Switzerland; ^9^Center for Medical Image Analysis and Navigation, Department of Biomedical Engineering, Faculty of Medicine, University of Basel, Allschwil, Switzerland

**Keywords:** multiple sclerosis, deep learning, advanced quantitative diffusion MRI, relative importance order, clinically correlated measure selection

## Abstract

Conventional magnetic resonance imaging (cMRI) in multiple sclerosis (MS) patients provides measures of focal brain damage and activity, which are fundamental for disease diagnosis, prognosis, and the evaluation of response to therapy. However, cMRI is insensitive to the damage to the microenvironment of the brain tissue and the heterogeneity of MS lesions. In contrast, the damaged tissue can be characterized by mathematical models on multishell diffusion imaging data, which measure different compartmental water diffusion. In this work, we obtained 12 diffusion measures from eight diffusion models, and we applied a deep-learning attention-based convolutional neural network (CNN) (GAMER-MRI) to select the most discriminating measures in the classification of MS lesions and the perilesional tissue by attention weights. Furthermore, we provided clinical and biological validation of the chosen metrics—and of their most discriminative combinations—by correlating their respective mean values in MS patients with the corresponding Expanded Disability Status Scale (EDSS) and the serum level of neurofilament light chain (sNfL), which are measures of disability and neuroaxonal damage. Our results show that the neurite density index from neurite orientation and dispersion density imaging (NODDI), the measures of the intra-axonal and isotropic compartments from microstructural Bayesian approach, and the measure of the intra-axonal compartment from the spherical mean technique NODDI were the most discriminating (respective attention weights were 0.12, 0.12, 0.15, and 0.13). In addition, the combination of the neurite density index from NODDI and the measures for the intra-axonal and isotropic compartments from the microstructural Bayesian approach exhibited a stronger correlation with EDSS and sNfL than the individual measures. This work demonstrates that the proposed method might be useful to select the microstructural measures that are most discriminative of focal tissue damage and that may also be combined to a unique contrast to achieve stronger correlations to clinical disability and neuroaxonal damage.

## Introduction

Conventional magnetic resonance imaging (cMRI) in multiple sclerosis (MS) plays a major role in MS diagnosis, prognosis, and in the evaluation of patients’ therapeutic response (Rovira et al., 2015; Wattjes et al., 2015). However, the heterogeneity of focal MS lesions, the pathology in normal-appearing white and gray matter (NAWM and NAGM), and the specific damage to myelin and axons are largely overlooked by cMRI. Multishell diffusion-weighted imaging (mDWI) provides a way to further probe tissue damage and repair in MS patients (Schneider et al., 2017; Lakhani et al., 2020). mDWI measures signal changes that are related to the diffusion of water molecules within central nervous system (CNS) tissue (Novikov et al., 2019; Lakhani et al., 2020), which is constrained by the local microenvironment (Novikov et al., 2019). This enables diffusion measures of biophysical microstructure models derived from mDWI to decode the information specific to different water compartments (e.g., intra-axonal and isotropic compartments) within the CNS tissue (Novikov et al., 2019). The intra-axonal compartment reflects the integrity of the neurites, and the isotropic compartment indicates the movement of the free water (Novikov et al., 2019). These two compartments can describe the two pathological presentations of MS lesions, demyelination, and axonal injury and are commonly modeled by various biophysical microstructure models (Lakhani et al., 2020).

A microenvironment characteristic is measured differently by the measures from different mathematical models due to the different assumptions on the diffusion within the tissue. Yet, to our knowledge, the direct comparison of all considered diffusion measures on MS lesions and the possibility to combine them does not exist. Therefore, how to select the most discriminating diffusion measures for a given neurological disorder and how to combine the complementary information they might provide remain to be open questions and motivate this study.

Convolutional neural network (CNN) in deep learning has proven to be promising in various applications of MR images and is able to encode spatial patterns on the images into representative hidden features (Andermatt et al., 2018; Yoo et al., 2018; Akçakaya et al., 2019; La Rosa et al., 2020; Saha et al., 2020). In our previous work (Lu et al., 2020), we used an attention-based CNN—GAMER-MRI—to rank the importance of the input quantitative MRIs in the classification of stroke and MS lesions. Here, we further developed the method to select discriminating intercorrelated diffusion measures in the classification of MS lesions and the perilesional tissue. Compared to the conventional feature selection methods, this CNN-based method enables utilizing maximally available spatial information of the images and does not need to decide on how to find representative values for the samples of each contrasts, such as the mean value only within a lesion neglecting the perilesion tissue. In addition, the method jointly considers all the contrasts, which is a limitation for most of the conventional feature selection methods. Furthermore, in this study, we have explored the relationship between the chosen measures, or their combinations, with the Expanded Disability Status Scale (EDSS) and the neurofilament light chain in the serum (sNfL), which are respectively (i) a clinical measure of disability in MS patients and (ii) a biological measure of neuroaxonal damage (Barro et al., 2018; Siller et al., 2019).

## Materials and Methods

### MRI Data

One hundred twenty-three MS patients (84 relapsing–remitting and 39 progressive, 71 female and 52 male, age range = 44.7 ± 14.0, median EDSS = 2.5, EDSS range of 0.0–8.0) were enrolled in the study, which was approved by the local Ethics Committee of Basel University Hospital. All subjects gave written consent prior to the enrollment. MS patients underwent a multiparametric protocol on 3T whole-body MR system (Siemens MAGNETOM Prisma). The protocol included 3D SPACE-based FLAIR, 3D magnetization-prepared 2 rapid gradient echoes (MP2RAGE) (Marques et al., 2010), and mDWI (Table 1).

**TABLE 1:** Acquisition parameters of each contrast in the MS dataset.

	TE (ms)	TR (ms)	FOV (mm^3^)	SR (mm^3^)	TI (ms)	Additional parameters
**FLAIR**	386	5000	256 × 256 × 256	l × l × l	1800	–
**MP2RAGE**	3	5000	256 × 256 × 256	l × l × l	700, 2500	–
						**b values (s/mm^2^)**
**mDWI**	75	4500	256 × 256 × 144	1.8 × 1.8 × 1.8	–	0/12 acquisitions and 12 reverse encoding acquisitions;
						700; 1000; 2000; 3000/137 directions in total

*TE, echo time; TR, repetition time; TI, inversion time; FOV, field of view; SR, spatial resolution.*

**FIGURE 1:**
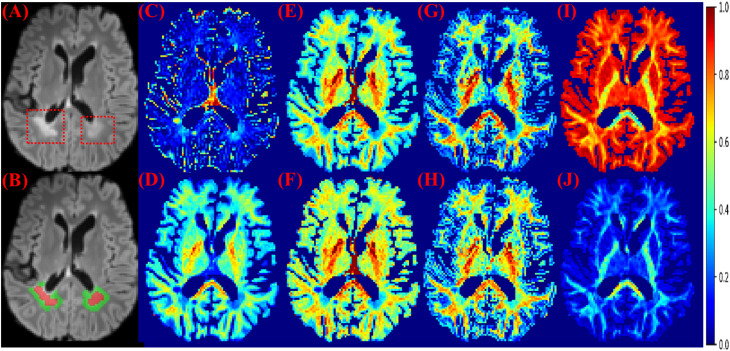
MS lesions on FLAIR and diffusion measures. **(A)** FLAIR: MS lesions are hyperintense and indicated by red dashed boxes. **(B)** Red: lesions; Green: perilesional white matter tissue. **(C)** The isotropic compartment from MB. **(D)** The intra-axonal compartment from MB. **(E)** The neurite density index from NODDI. **(F)** The intra-axonal compartment from SMT-NODDI. **(G)** The intra-axonal compartment from MCMDI. **(H)** The intra-axonal compartment from NODDIDA. **(I)** The isotropic compartment from Ball and Stick. **(J)** The intra-axonal compartment from Ball and Stick. Other measures in the analysis are in Supplementary Figure 1.

Measured diffusion-weighted imaging was denoised by MRtrix (Cordero-Grande et al., 2019; Tournier et al., 2019). The correction of susceptibility-induced distortion with the reversed phase-encoding images, eddy currents, and movement was performed by FMRIB Software Library (FSL) (Andersson et al., 2003; Smith et al., 2004; Jenkinson et al., 2012; Andersson and Sotiropoulos, 2016). The quantitative diffusion measures for the isotropic and intra-axonal compartments were reconstructed from the eight open-source biophysical models, including Ball and Stick^1^ (Behrens et al., 2003), neurite orientation and dispersion density imaging (NODDI)^2^ (Zhang et al., 2012), NODDI with the spherical mean technique (SMT-NODDI)^1^ (Cabeen et al., 2019), microstructure Bayesian (MB) approach^3^ (Reisert et al., 2017), multicompartment microscopic diffusion imaging (MCMDI)^1^ (Kaden et al., 2016), neurite orientation dispersion and density imaging with diffusivities assessment (NODDIDA)^4^ (Jelescu et al., 2015), distribution of 3D anisotropic microstructural environments in diffusion-compartment imaging (DIAMOND)^5^ (Scherrer et al., 2016), and microstructure fingerprinting^6^ (Rensonnet et al., 2019). The exemplary diffusion measures and FLAIR are in Figure 1.

The quantitative diffusion measures of each patient were masked by the brain mask to remove non-brain tissue including the ventricle. The brain mask was the binarized subcortical segmentation obtained from FreeSurfer (Fischl et al., 2001) on MP2RAGE (Fujimoto et al., 2014) and transformed by FSL to align with mDWI. The diffusion measures were then subject-wise normalized. Eighty-four patients were randomly selected to be used in a 5-fold cross-validation. The other 39/123 patients formed a pure test dataset. White matter lesions were automatically segmented using FLAIR and MP2RAGE^7^ (La Rosa et al., 2020) and manually corrected by two expert raters. The lesion segmentations were transformed by FSL to be aligned with mDWI. Lesions of size less than three voxels were excluded. The perilesional tissue was defined as white matter tissue locating within a three-voxel region around the lesions. Patches of 5 × 5 × 5 voxels were sampled on lesions and perilesional tissue considering the lesion sizes. To reduce the overlapping between the lesion and perilesional patches due to their proximity, a constraint of at most 20% of a sampled patch being overlapped with another patch was applied. The numbers of patches being sampled on each lesion and perilesional tissue were proportional to the size of the lesion and the perilesional tissue, respectively. In the end, 3007 lesion patches and 3624 perilesional patches were sampled in the dataset for 5-fold cross-validation, and 1402 lesion patches and 1665 perilesional patches were sampled in the pure test dataset. The 5-fold cross-validation was based on the number of patients. Therefore, patches from a patient would not present both in the training and in the validation datasets.

### GAMER-MRI

GAMER-MRI was previously developed and validated as a method to obtain attention weights and the relative importance in a classification task of given input contrasts (Lu et al., 2020). As we previously reported, the neural network consisted of three parts for feature extraction, gated attention mechanism (Ilse et al., 2018), and classification (Lu et al., 2020). The feature extraction part included three convolutional blocks for each contrast. Each convolutional block was composed of a layer of 16 convolutional filters and exponential leaky units followed by batch normalization. The kernel size of the convolutional filter was 3 × 3 × 3, and padding was applied correspondingly to maintain the patch size. After the last convolutional block, a 16-neuron fully connected layer (FCL) received the flattened vector of 125 elements and encoded the hidden feature of 16 elements. The gated attention mechanism was formed by an attention layer containing an eight-neuron FCL followed by the tanh function and a gate layer having an eight-neuron FCL followed by the sigmoid function. The outputs of tanh and sigmoid were element-wise multiplied. From the element-wise product, in the original implementation for not-highly-correlated input contrasts, the attention weights were obtained by following one-neuron FCL and the softmax function (Lu et al., 2020). However, this design was not effective for highly correlated inputs, i.e., diffusion measures in this work. The information content of measures is similar, and thus, the difference in the obtained attention weights was small.

**FIGURE 2:**
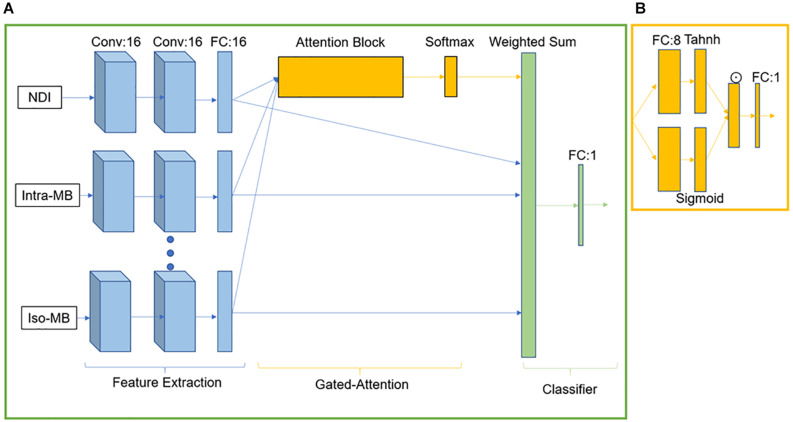
GAMER-MRI. **(A)** The neural network. Conv stands for the convolutional block. FC is a fully connected layer. **(B)** Attention block. ☉ represents an element-wise multiplication.

For the purpose of this study, we multiplied the outputs from the element-wise multiplication by 2. This enhanced the difference between the encoded features of the correlated diffusion measures during training because the exponential transformation in the softmax function could not properly reflect the difference in the small and negative values. For example, 0.01 is 10 times larger than 0.001, but they become 1.01 and 1.001 after the exponential transformation. This leads to 0.502 and 0.498 as attention weights after the softmax function. The enhanced output was then connected to a one-neuron FCL followed by the softmax function to generate the normalized attention weights. The weighted sum of the hidden features and the corresponding attention weights formed a combined hidden feature for the classifier. The classifier was one sigmoid neuron. The network structure is in Figure 2.

The weighted sampler was used to account for the class imbalance, and the batch size was 256. The loss function was cross-entropy loss. The evaluation metric was the area under the receiver operating characteristic curve (AUC). The optimizer was AdamW (Loshchilov and Hutter, 2019) with the learning rate = 5e-5 and the weight decay = 1e-2. To avoid overfitting, data augmentation and a learning-rate scheduler were performed. On-the-fly data augmentation included random flipping in the left–right directions and Gaussian noise with zero mean and unit standard deviation. The scheduler was the learning-rate-reduce-plateau scheduler with a patience of 15 epochs.

### Selection of Contrasts

Intrinsic strong correlation between the quantitative diffusion measures can lead to instability of the obtained attention weights and the ranked order, compared to the result in Lu et al. (2020). Therefore, to avoid determination solely based on the attention weights, the selection of discriminating measures was an iteration process. It started from the measure whose attention weight was dominant in the validation datasets in all the cross-validation folds. If no measure was selected, the measures whose attention weights were ranked first or second in all the folds were considered. If no measures were selected, the attention weights that ranked first or second and third in all the folds were considered. The selection stopped when the sum of their attention weights was over 0.5, which meant that the selected measures were more important than 50% of the input diffusion measures in differentiating the lesion and perilesional tissue.

**FIGURE 3:**
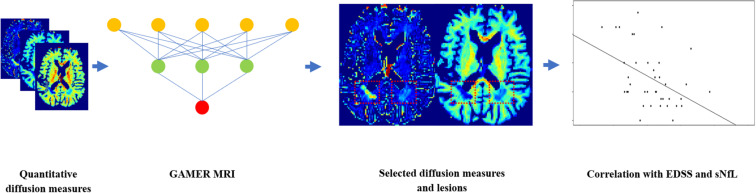
Flowchart for using GAMER-MRI to select the most discriminating subject-wise normalized diffusion measures and correlating the combinations of selected diffusion measures with the Expanded Disability Status Scale and the serum level of neurofilament light chain.

To assess which selected subject-wise normalized quantitative diffusion measures, or combination of those measures, was best correlated with patients’ EDSS as well as NfL in the pure test dataset, we first averaged the diffusion measures within each lesion and then over lesions within each patient. In 31/39 patients of the test dataset, we quantified sNfL. Then, we performed Spearman’s correlation coefficient with two-sided 20,000 permutation tests. The Benjamin–Hochberg procedure (Benjamini and Hochberg, 1995) was performed to control the false discovery rate (FDR) with the threshold 0.05. The flowchart is shown in Figure 3.

## Results

### Lesion Classification

In Table 2, we report the average performance of GAMER-MRI using all the diffusion measures on the (i) validation dataset over fivefold cross-validation and (ii) on the pure test dataset.

**TABLE 2:** Performance of the patch-based network on MS lesions and the selected diffusion measures on fivefold cross-validation (first row, average mean, and standard deviation are reported) and pure testing set (second row). Balanced accuracy is defined as the average of sensitivity and specificity in each fold. Fl score is defined as the harmonic mean of precision and recall.

Mean metrics (%)	AUC	Balanced accuracy	Sensitivity	Specificity	F1 score
Validation dataset	90.67 ± 0.009	83.26 ± 1.35	81.09 ± 2.44	85.44 ± 2.03	81.62 ± 1.67
Test dataset	91.01 ± 0.003	83.42 ± 0.12	83.39 ± 0.67	83.45 ± 0.82	82.14 ± 0.11

**Selected measures**	**NDI**	**Intra-MB**	**Iso-MB**	**Intra-SMT**	

Attention weights	0.121 ± 0.014	0.117 ± 0.014	0.145 ± 0.007	0.131 ± 0.015	

The diffusion measures selected by using the validation datasets were the neurite density index (NDI) from NODDI, the intra-axonal and isotropic compartment from MB (Intra-MB and Iso-MB), and the intra-axonal compartment from SMT-NODDI (Intra-SMT) in Figure 1. Their average attention weights of the corrected predicted samples are also reported in Table 2.

### Spearman’s Correlation

#### Correlation With EDSS

**FIGURE 4:**
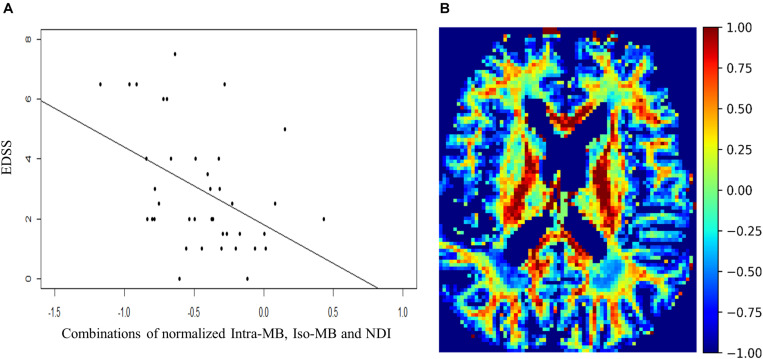
**(A)** Scatter plot and a regression line of EDSS and the combinations of normalized Intra-MB, Iso-MB, and NDI, which has strongest correlation. **(B)** An exemplary image of the combined contrast.

The Spearman’s correlation coefficients (ρ) and the corresponding original *p*-values of the selected normalized diffusion measures, or their statistically significant combinations and EDSS, are reported in Table 3. The Spearman’s correlation coefficients (ρ) of the conventional lesion load metrics are also reported. The number of potential combinations of four selected diffusion measures is 15, and there are two tests in the lesion load analysis. This led to in total 17 statistical tests. The significance controlled by FDR is indicated by an asterisk. The scatter plot of the combination having the strongest correlation is in Figure 4A, and an exemplary image of the combination is in Figure 4B.

**TABLE 3:** Spearman’s correlation of selected normalized diffusion measures, or their combinations and EDSS.

Lesion load	ρ	*P*-value	Significance
Number of lesions	0.13	0.41	–
Lesion volume	0.25	0.12	–
**Normalized diffusion measures**			
NDI	−0.38	0.017	*
Intra-SMT	−0.31	0.057	
Intra-MB	−0.40	0.013	*
Iso-MB	0.09	0.58	–
Intra-MB + Iso-MB	−0.39	0.014	*
Intra-MB + NDI	−0.43	0.007	*
Intra-SMT + NDI	−0.37	0.023	*
Intra-SMT + Intra-MB	−0.40	0.012	*
Intra-MB + Iso-MB + NDI	−0.45	0.004	*
Intra-MB + Iso-MB + Intra-SMT	−0.42	0.007	*
Intra-MB + Intra-SMT + NDI	−0.42	0.009	*
Intra-MB + Iso-MB + NDI + Intra-SMT	−0.41	0.009	*

*The significance is controlled by FDR with a threshold of 0.05. Only the combinations of significance are reported.*

#### Correlation With sNfL

**FIGURE 5:**
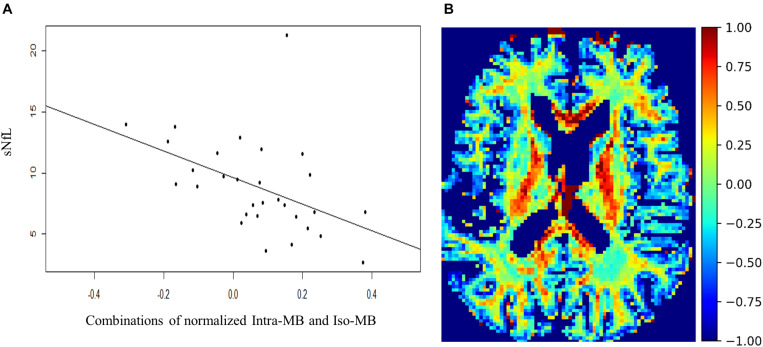
**(A)** Scatter plot and a regression line of the combinations of normalized Intra-MB and Iso-MB, which showed the strongest correlation with sNFL. **(B)** An exemplary image of the combined contrast.

The Spearman’s correlation coefficients (ρ) and the corresponding original *p*-values are reported in Table 4. One patient had a relatively high sNfL level of 160 μg/ml, compared to the mean sNfL level of 8.9 μg/ml of the rest of 30 patients. After this patient’s data were excluded, the significance in Table 4 did not change, but the correlation was stronger. For illustration purpose, the scatter plot of the combination having the strongest correlation (Figure 5A) does not contain this outlier patient. An exemplary image of the combination is in Figure 5B.

**TABLE 4:** Spearman’s correlation of selected normalized diffusion measures, or their combinations and sNfL.

Lesion load	ρ	*P*-value	Significance
Number of lesions	0.48	0.006	*
Lesion volume	0.45	0.01	*
**Normalized diffusion measures**			
NDI	−0.37	0.04	–
Intra-SMT	−0.27	0.14	–
Intra-MB	−0.42	0.02	*
Iso-MB	0.1	0.59	–
Intra-MB + Iso-MB	−0.51	0.004	*
Intra-MB + NDI	−0.43	0.02	*
Intra-MB + Iso-MB + NDI	−0.48	0.007	*
Intra-MB + Iso-MB + Intra-SMT	−0.45	0.01	*
Intra-MB + Iso-MB + NDI + Intra-SMT	−0.44	0.02	*

*The significance controlled by FDR with a threshold of 0.05. Only the combinations of significance are reported.*

## Discussion

Our work provided evidence that a modified version of GAMER-MRI, including a specific selection procedure for correlated measures, permits to identify the microstructural diffusion measures that are most discriminative of focal MS pathology among the ones obtained with eight open-source mathematical models of multishell diffusion data. Moreover, our data showed that some of the combinations of the selected normalized diffusion measures better correlated with patients’ disability and neuroaxonal damage than the individual measures.

Diffusion-based microstructural measures quantify different compartments based on various assumptions. Nevertheless, the relative sensitivity of the different diffusion-based microstructural metrics to specific CNS pathologies is unclear. In this work, we have provided a methodological frame to discriminate the most sensitive diffusion microstructural measures to focal MS pathology in a large population of MS patients.

We first aimed at identifying which measure best discriminated MS lesions from the perilesional tissue because we judged that if the neural network was able to differentiate between lesions and the immediate surrounding tissue, the learned pattern would have been most sensitive to focal MS pathology than the one we would have derived by comparing lesions to the distant normal-appearing tissue. The evaluation metrics in Table 2 indicated that the neural network was able to learn pivotal information for the target classification. As expected, because of the highly correlated nature of the studied diffusion-based measures, the difference among the obtained attention weights was small. The proposed selection process alleviated the fluctuating order of attention weights due to their small differences. The threshold of 0.5 in the selection process was empirically chosen considering the representativeness of selected diffusion measures and the multiple comparison problem.

The core idea of the attention mechanism is to enhance important features from the data themselves relevant to the specific application (Bahdanau et al., 2015). Therefore, in most of the applications in natural language processing and natural image classification, the attention weights were used to enhance the connection to the corresponding features based on their importance instead of quantifying the relative importance among the features (Maicas et al., 2017; Vaswani et al., 2017; Hu et al., 2018; Woo et al., 2018). Using different designs of the attention mechanism, the attention weights also provide the relative importance among features as shown in a histopathological image classification and image captioning (You et al., 2016; Ilse et al., 2018). In GAMER-MRI, attention weights were computed and validated on multicontrast MRI measures in order to select their relative importance in a given neurological disease classification.

To our knowledge so far, only few studies applied measures derived from microstructural models to study focal MS pathology (for a review, see Granziera et al., 2020) and only one study used deep-learning to show the superior performance of diffusion basis spectrum imaging to segment voxel-wise different types of MS lesions compared to using diffusion tensor imaging (Ye et al., 2020). However, the joint comparison of multiple microstructural diffusion measures in MS lesions has not been explored yet. This work considered the potential interaction between the measures and tried to address this issue.

The four selected diffusion measures include three measures for the intra-axonal compartment from three models and one measure for the isotropic compartment from one of the three models. This means that most of the discriminating information of the damaged neurons was from the loss of axonal integrity. The additional information about the inflammatory processes might be reflected by the measure for the isotropic compartment to better characterize the distinction of lesions.

Besides, by combining the selected diffusion measures in the discrimination of focal pathology, it was possible to achieve a stronger correlation with patient disability than one of those metrics alone or even conventional MRI metrics, such as the lesion number and volume. These results suggest that a comprehensive description of the tissue microstructure in regions of focal damage in MS patients may well help decrease the clinical–radiological paradox (Barkhof, 2002). Interestingly, the combined contrast achieving the best correlation with disability was the sum of measures quantifying intra-axonal and isotropic diffusion, which may be considered surrogate measures of the loss of integrity of axons and myelin as well as of inflammatory processes (i.e., increased cellularity and edema).

Most of the combinations that best correlated to EDSS were also highly related to the sNfL levels: remarkably, the correlation coefficients between sNfL and combinations of diffusion-MRI metrics were even higher than the ones obtained between sNfL and the lesion load, which is known to be highly related to sNfL levels (Chitnis et al., 2018; Todea et al., 2020). The patient, who had an extremely high level of sNfL, had a relapse 2 months before the sNfL acquisition, which may have well influenced the strong increased in sNfL levels.

To perform the correlation analyses with EDSS and sNFL, we have used subject-wise normalized maps of diffusion-based microstructural measures, which were the ones encoded by GAMER-MRI. We also trained the neural network on the original images, which, however, led to worse classification performance. Because subject-wise normalized maps were used, it is challenging to determine whether the network could learn the right pattern and to generate representative attention weights. Owing to the applied normalization procedure, the interpretation of the pathological meaning of the combined metrics is particularly difficult. Another limitation of this study was that we divided the cross-validation folds based on the number of patients instead of the number of patches: this led to different distributions of lesion and perilesional patches in the validation datasets of all cross-validation folds and to the fluctuation of the validation results. On the other hand, this also had the advantage of preventing the leak of information induced by the appearance of patches from one patient in both the training and validation dataset. Based on the obtained result (Table 2), the performance on the test dataset was stable, so the limitation was alleviated.

## Conclusion

In summary, our work showed that the proposed attention-based neural network and the selection process based on the previous work can select important diffusion measures despite that they are highly intercorrelated. Those measures have the potential to be combined to enhance the correlation with the clinical measures. Future work will be required to directly find the best combinations without using a statistical test and tackling the multiple comparison problem. Furthermore, the use of a combination of diffusion-based microstructural measures deserves further attention and development, allowing a better interpretability of its pathological meaning.

## Data Availability Statement

The original contributions presented in the study are included in the article/Supplementary Material, further inquiries can be directed to the corresponding author/s.

## Ethics Statement

The studies involving human participants were reviewed and approved by the local Ethics Committee of Basel University Hospital. The patients/participants provided their written informed consent to participate in this study.

## Author Contributions

P-JL: conceptualization, data curation, methodology, investigation, formal analysis, and writing—original draft. MB: data curation, methodology, and writing—reviewing and editing. MW: resources, data curation, and writing—reviewing and editing. RR, RG, and FL: data curation and writing—reviewing and editing. SS, MBC, and AD: resources and writing—reviewing and editing. RS: conceptualization and writing—reviewing and editing. JK and LK: writing—reviewing and editing. PC: supervision and writing—reviewing and editing. CG: supervision, conceptualization, funding acquisition, resources, and writing—reviewing and editing. All authors contributed to the article and approved the submitted version.

## Conflict of Interest

The authors declare that the research was conducted in the absence of any commercial or financial relationships that could be construed as a potential conflict of interest.

## Footnotes

1 https://github.com/AthenaEPI/dmipy

2 https://github.com/daducci/AMICO

3 https://bitbucket.org/reisert/baydiff/wiki/Home

4 https://github.com/robbert-harms/MDT

5 https://bitbucket.org/benoitscherrer/crldciestimate

6 The author needs to be contacted.

7 https://hub.docker.com/r/francescolr/ms_seg
