# Supplementary material for: GAMER-MRI in Multiple Sclerosis Identifies the Diffusion-Based Microstructural Measures That Are Most Sensitive to Focal Damage: A Deep-Learning-Based Analysis and Clinico-Biological Validation
Source: Front Neurosci. 2021 Apr 6;15:647535. doi: 10.3389/fnins.2021.647535 (PMC8055933; doi:10.3389/fnins.2021.647535)
Supplement: Supplementary file 1 [file Table_1.DOCX]

**Supplementary Materials:**


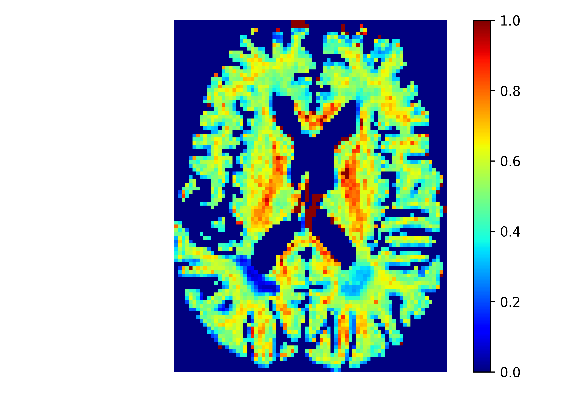

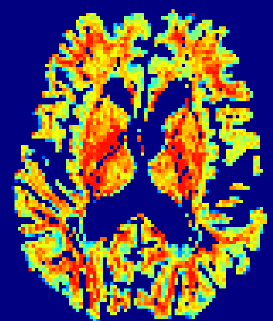

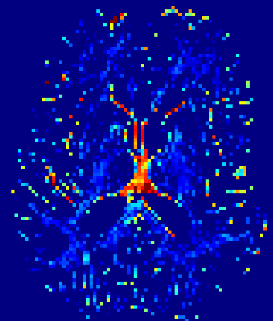

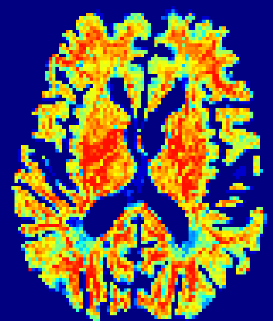

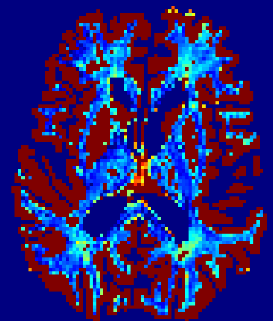


**Supp. Figure 1:** Other diffusion measures in the analysis. (A) The first intra-axonal compartment from microstructure fingerprinting (MF-f0). (B) The second intra-axonal compartment from microstructure fingerprinting (MF-f1). (C) The intra-axonal compartment from DIstribution of 3-D Anisotropic MicrOstructural eNvironments in Diffusion-compartment imaging (DIAMOND). (D) The isotropic compartment from Neurite Orientation and Dispersion Density Imaging (ISOVF)

**(A)**

**(B)**

**(C)**

**(D)**

| Diffusion Measures | NDI | Intra-MB | Iso-MB | Intra-SMT | ISOVF | Intra-MCMDI |
| --- | --- | --- | --- | --- | --- | --- |
| Attention Weights | 0.121±0.014 | 0.117±0.014 | 0.145±0.007 | 0.131±0.015 | 0.038±0.014 | 0.098±0.010 |
| Diffusion Measures | **Intra-Ball and Stick** | **Iso-Ball and Stick** | **Intra-DIAMOND** | **Intra-NODDIDA** | **MF-f0** | **MF-f1** |
| Attention Weights | 0.049±0.012 | 0.055±0.005 | 0.030±0.012 | 0.049±0.007 | 0.090±0.001 | 0.077±0.004 |

**Supp. Table 1:** The attention weights of the diffusion measures in the validation dataset on 5-fold cross-validation (average mean and standard deviation are reported). The prefix, “intra-“, stands for the intra-axonal compartment of the following model. The prefix. “iso”, represents the isotropic compartment of the following model.
